# Importance and Management of Non–High-Density Lipoprotein Cholesterol in Dyslipidemia Treatment

**DOI:** 10.1016/j.jacasi.2025.12.024

**Published:** 2026-04-07

**Authors:** Richard C. O’Brien, Lourdes Ella Gonzalez-Santos, Brian Tomlinson, Zanariah Hussein, Soo Lim, Hapizah Nawawi, Hean Yee Ong, Silki Silki, Sidartawan Soegondo, Ta-Chen Su, Apichard Sukonthasarn, Pham Nguyen Vinh

**Affiliations:** a Melbourne Medical School, University of Melbourne, Melbourne, Victoria, Australia; b Austin Health, Heidelberg, Austrailia; c University of the Philippines—Philippine General Hospital, Manila, Philippines; d Faculty of Medicine, Macau University of Science and Technology, Macau SAR, China; e Medical Department, Hospital Putrajaya, Putrajaya, Malaysia; f Department of Internal Medicine, Seoul National University College of Medicine and Seoul National University Bundang Hospital, Seongnam, South Korea; g Hospital Al-Sultan Ahmad, Universiti Teknologi MARA, Puncak Alam, Shah Alam, Malaysia; h Cardiac Solutions Medical Centre, Singapore; i Medical Affairs, Indegene Limited, Bangalore, India; j Diabetes Connection and Care EKA Hospitals, and Indonesia Diabetes Institute, Jakarta, Indonesia; k Departments of Internal Medicine and Environmental and Occupational Medicine, National Taiwan University Hospital and College of Medicine, National Taiwan University, Taipei, Taiwan; l Faculty of Medicine, Chiang Mai University, Chiang Mai, Thailand; m Cardiovascular Center, Tam Anh General Hospital, Ho Chi Minh City, Vietnam

**Keywords:** APAC consensus, atherogenic dyslipidemia, fibrates, modified Delphi, non-HDL-C, residual risk

## Abstract

Residual cardiovascular (CV) risk persists despite achieving low-density lipoprotein cholesterol (LDL-C) targets with lipid-lowering therapy, indicating the role of other lipid parameters. Robust evidence has shown that non-high-density lipoprotein cholesterol (non-HDL-C) confers better estimation of atherosclerotic cardiovascular disease risk than LDL-C and is increasingly recognized as a treatment target in atherogenic dyslipidemia. Whereas global guidelines have incorporated non-HDL-C as a primary or co-primary lipid target, most Asia-Pacific region (APAC) guidelines still consider it secondary to LDL-C. To address this gap, we developed a consensus document with evidence-based recommendations with the use of a modified Delphi method involving 11 experts across 10 APAC countries. This offers practical guidance on adopting non-HDL-C as a lipid target, including add-on therapies to statins for reducing non-HDL-C and CV risk in atherogenic dyslipidemia. Acknowledging regional variations in lipid targets, this document does not override local APAC guidelines but rather encourages physicians to manage non-HDL-C in alignment with those guidelines.

## Highlights

• Residual cardiovascular risk persists despite achieving LDL-C targets, highlighting the need for additional lipid markers.
• Non-HDL-C is recommended as a primary or co-primary lipid target for better ASCVD risk assessment, especially in atherogenic dyslipidemia.
• Management strategies include intensifying LDL-lowering therapies and adding fibrates or omega-3 fatty acids to reduce non-HDL-C and CV risk.
• Future research should focus on clinical implementation strategies for non-HDL-C targets across the Asia-Pacific region.

Lowering of low-density lipoprotein cholesterol (LDL-C), a long-established risk factor for atherosclerotic cardiovascular disease (ASCVD),^1^ with statin therapy remains the cornerstone of cardiovascular disease (CVD) risk reduction.^2^, ^3^, ^4^, ^5^, ^6^, ^7^, ^8^, ^9^ However, residual cardiovascular (CV) risk persists in patients whose LDL-C levels are at goal, indicating that additional factors beyond LDL-C contribute to the residual risk.^10^, ^11^, ^12^, ^13^, ^14^ Evidence suggests that elevated triglycerides (TGs), very-low-density lipoprotein (VLDL), intermediate-density lipoprotein (IDL), chylomicron remnants, and lipoprotein (a) (Lp(a)) also contribute to atherogenicity and increase the CV risk regardless of LDL-C levels.^15^^,^^16^ Robust evidence has shown that non-high-density lipoprotein cholesterol (non-HDL-C) and apolipoprotein B (ApoB), as measures of all atherogenic particles, confer better estimation of ASCVD risk than does LDL-C.^17^, ^18^, ^19^, ^20^, ^21^, ^22^ The cost and limited availability of ApoB testing, however, are factors in its use as a marker.^15^^,^^23^ In contrast, non-HDL-C is easily calculated and incurs no additional costs above the standard lipid profile,^23^ making it an emerging treatment target in atherogenic dyslipidemia.^3^

Atherogenic dyslipidemia, characterized by elevated TGs and low levels of high-density lipoprotein cholesterol (HDL-C), often along with elevated ApoB and non-HDL-C, is common in patients with CVD, type 2 diabetes mellitus (T2DM), obesity, and metabolic syndrome (MetS) and is associated with macro- and microvascular residual risks.^3^^,^^24^^,^^25^ A recent analysis suggested that from 1980 to 2018, the trend of elevated non-HDL-C levels shifted from Western European countries to those in Asia and the Pacific (including Malaysia, the Philippines, and Thailand).^26^ This trend corroborates the wide prevalence of hypertriglyceridemia, low HDL-C,^27^^,^^28^ obesity, MetS, and T2DM reported in the Asia-Pacific (APAC) region^29^, ^30^, ^31^, ^32^ and underscores the need to manage rising non-HDL-C levels to reduce the residual ASCVD risk.

The introduction of non-HDL-C as a co-primary or primary lipid target in major global guidelines highlights its importance in dyslipidemia and CVD management.^5^^,^^33^, ^34^, ^35^, ^36^ However, most APAC guidelines still focus on LDL-C as the primary target, with non-HDL-C as the secondary target (Table 1).

**Table 1:** Non-HDL-C Recommendations in Europe and Asia-Pacific Guidelines

	Country	Author, Year	Recommendation on Non-HDL-C	Primary or Secondary Target; Target Value if Available
1.	Europe: European Society of Cardiology and European Atherosclerosis Society for the Management of Dyslipidaemias	Mach et al, 2019^3^	Recommended for risk assessment, particularly in people with high TG levels, diabetes mellitus, obesity, or very low LDL-C levels	Secondary goal: 0.8 mmol/L (30 mg/dL) higher than the corresponding LDL-C goal; non-HDL-C secondary goals are <2.2, <2.6, and <3.4 mmol/L (<85, <100, and <130 mg/dL) for very high, high, and moderate risk people, respectively
2.	China: China Guidelines for the Management of Blood Lipids	Li et al, 2023^37^	Recommended as a secondary target of intervention against ASCVD	Secondary target: LDL-C + 0.8 mmol/L
3.	India: Cardiological Society of India Clinical Practice Guidelines for Dyslipidemia Management	Sawhney et al, 2023^38^	Recommended as a co-primary goal, stratified according to CV risk categories	Co-primary goal: 30 mg/dL higher than recommended LDL-C target; non-HDL-C: <70, <85, <100, <130, and <130 mg/dL for extremely high, very high, high, moderate, and low risk groups
4.	Taiwan: Guidelines of the Taiwan Society of Cardiology on the Primary Prevention of Atherosclerotic Cardiovascular Disease	Chao et al, 2024^39^	Recommended to be used to predict the risk of ASCVD, especially in patients with elevated TG, diabetes mellitus, and obesity, in whom LDL-C measurement may be underestimated	Secondary target: 30 mg/dL above the recommended LDL-C target
5.	Japan: Japan Atherosclerosis Society Guidelines for Prevention of Atherosclerotic Cardiovascular Diseases	Okamura et al, 2022^6^	Recommended as a secondary target for prevention of ASCVD	Secondary target: 30 mg/dL above LDL target
6.	South Korea: Guidelines for the Management of Dyslipidemia in Korea	Rhee et al, 2018^9^	Recommended as secondary goal after achieving the targeted LDL-C concentration based on CVD risk factors	Secondary goal: 30 mg/dL above the recommended LDL-C target; non-HDL-C: <100, <130, <160, and <190 mg/dL for very high, high, moderate, and low risk groups
7.	Singapore: Agency for Care Effectiveness Clinical Guidance: Lipid Management	Agency for Care Effectiveness, 2023^40^	NA	NA
8.	Philippines: Clinical Practice Guidelines for the Management of Dyslipidemia	Gonzalez-Santos et al, 2020^41^	Recommended as an additional therapeutic target to better estimate of CV risk beyond LDL-C, especially in patients with metabolic syndrome, type 2 diabetes mellitus, and obesity	Additional target: 30 mg/dL above target LDL-C
9.	Malaysia: Clinical Practice Guidelines on the Management of Dyslipidemia	National Heart Association of Malaysia, 2023^42^	May be considered as a secondary target when treating patients with combined hyperlipidemia, diabetes, cardiometabolic risk, and chronic kidney disease; in cases with TG >4.5 mmol/L, non-HDL-C becomes the primary target of therapy	Secondary target: 0.8 mmol/L higher than the corresponding LDL-C target level
10.	Thailand: Clinical Practice Guidelines on Management of Dyslipidemia for Atherosclerotic Cardiovascular Disease Prevention	Lolekha et al, 2024^43^	Recommended to calculate non-HDL-C in individuals with high TG levels, diabetes, or obesity	NA
11.	Sri Lanka: National Guidelines for Management of Dyslipidemia for Primary Health Care Providers	Directorate of Non-communicable Disease, 2021^44^	NA	NA
12.	Australia: Australian Guideline for Assessing and Managing Cardiovascular Disease Risk	National Heart Foundation of Australia, 2023^45^	Non-HDL-C and ApoB levels are better predictors of CV events than LDL-C	NA

ApoB = apolipoprotein B; ASCVD = atherosclerotic cardiovascular disease; CV = cardiovascular; CVD = cardiovascular disease; HDL-C = high-density lipoprotein cholesterol; LDL-C = low-density lipoprotein cholesterol; TG = triglyceride.

Because many physicians within APAC still focus on managing the primary target in clinical practice, this may lead to suboptimal CV risk reduction for dyslipidemic patients. Thus, there is a need for more emphasis on non-HDL-C as an additional treatment target and its clinical application in the APAC region. The present consensus statement proposes evidence-based recommendations for using non-HDL-C as a treatment target in dyslipidemia management, developed through opinions of clinicians across 10 APAC countries. These expert recommendations cover various aspects of non-HDL-C, such as its importance, use as a dyslipidemia treatment target, and management, particularly, fibrate therapy. Importantly, this consensus does not recommend specific levels of non-HDL-C, because lipid targets vary across guidelines of different countries. However, we think that there should be more emphasis on non-HDL-C for CV risk reduction than previously, and therefore provide practical guidance for managing non-HDL-C in a manner consistent with local lipid guidelines.

## Methods

**Figure 1:**
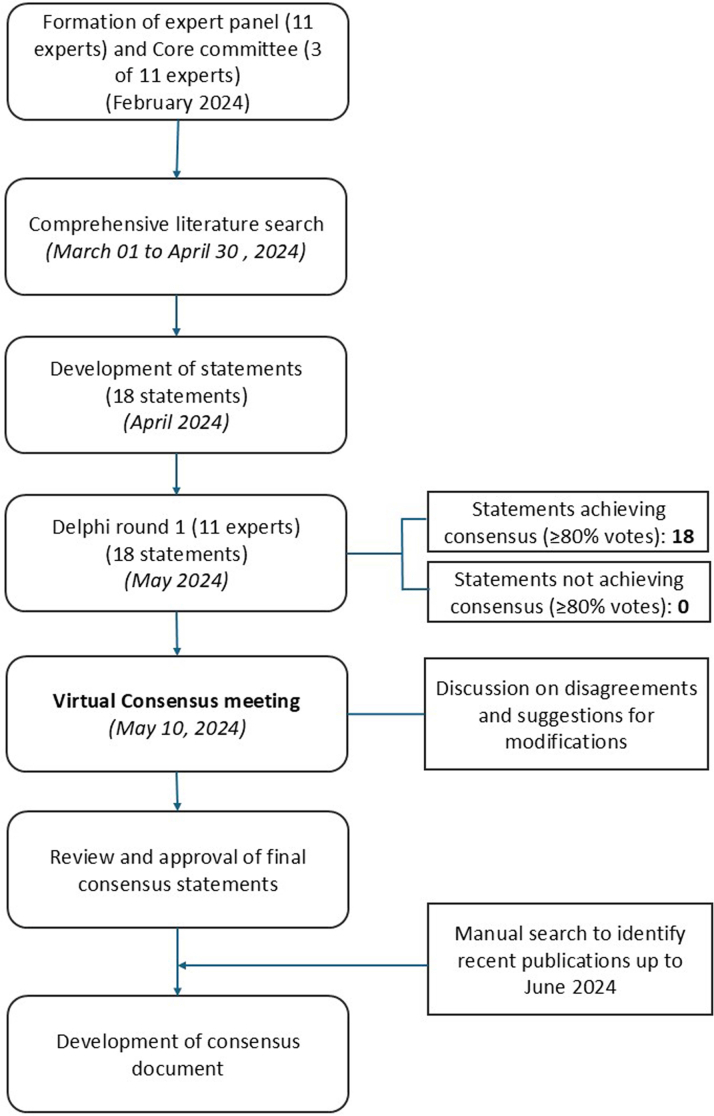
Process Flow for the Development of a Consensus Document With the Use of the Modified Delphi Method The figure outlines key steps in the development of consensus statements, including formation of a core committee, literature search, and development of the statements; selection of a panel comprising experts of the Asia-Pacific region; voting on the statements by the panel; discussion of the voting results; and finalization of the consensus statements by the expert panel.

The study used a modified Delphi method (1 round of online voting followed by a virtual meeting) to obtain responses to questions about the importance of non-HDL-C, its application as a treatment target for dyslipidemia, and its management. Details of the methodology, as outlined in Figure 1, are provided in the Supplemental Methods.

A comprehensive literature search was performed from March 1 to April 30, 2024, in the PubMed database with the use of specific keywords (Supplemental Table 1). This was complemented with a manual search up to June 2024. Based on the literature and clinical evidence, the core group developed a set of 18 statements regarding importance of non-HDL-C (8 statements), non-HDL-C as a treatment target in dyslipidemia and its management (3 statements), and difference among various treatment options (7 statements) (Supplemental Table 2).

## Results

Here we report the final consensus statements and recommendations from the meeting of clinical experts regarding the importance of non-HDL-C and its management in the treatment of dyslipidemia and summarize the supporting literature. In the first round of voting, responses from all 11 experts were obtained, and all 18 proposed statements achieved consensus (≥80% agreement) (Figure 1). Ten statements achieved 100% agreement. Seven statements achieved 91%, and 1 statement achieved 82% agreement. Statements that received any disagreements or suggestions for modifications were discussed during the meeting and modified based on collaborative deliberations to achieve 100% agreement.

## Consensus Statements and Recommendations

### Residual risk despite achieving LDL-C goals

***Consensus statement 1:*** *Despite achieving the LDL-C target, the residual risk of ASCVD persists in patients with dyslipidemia, even after adjustment for other risk factors. This may be attributed to other atherogenic lipoproteins, including Lp(a) and TG-rich particles.*

LDL-C has been widely considered to be a major risk factor for ASCVD and a target for dyslipidemia management. Statins, which reduce LDL-C levels, have been the mainstay therapy for preventing CV events.^3^, ^4^, ^5^, ^6^, ^7^, ^8^, ^9^ However, large, randomized trials involving individuals with or without CVD support that the CV risk and mortality persist despite achieving LDL-C goals, highlighting the limitations of LDL-C in predicting ASCVD.^10^, ^11^, ^12^, ^13^^,^^46^ In the PROVE IT-TIMI 22 (Pravastatin or Atorvastatin Evaluation and Infection Therapy—Thrombolysis In Myocardial Infarction 22) trial, 4,162 patients with a history of acute coronary syndrome (ACS) achieved median LDL-C levels of 95 mg/dL (2.46 mmol/L) with standard therapy (40 mg of pravastatin) and 62 mg/dL (1.60 mmol/L) with intensive therapy (80 mg of atorvastatin; *P* < 0.001). However, despite achieving lower levels of LDL-C, the CV event rate remained high, even in the intensive treatment group: the Kaplan-Meier estimates of the primary endpoint at 2 years were 26.3% in the pravastatin group and 22.4% in the atorvastatin group.^12^ Similarly, the EMPATHY (Standard Versus Intensive Statin Therapy for Hypercholesterolemic Patients With Diabetic Retinopathy) study, involving patients without a history of coronary artery disease (CAD), reported a cumulative incidence of CV events or CV-associated deaths as 5.8% (standard group) and 5.4% (intensive group), despite achieving the mean LDL-C levels of 104.1 ± 22.1 and 76.5 ± 21.6 mg/dL (*P* < 0.001), respectively, at 36 months.^13^

Studies from APAC countries have reported similar findings. In an Indian case-control study, 64% (32/50) of patients with CAD had target LDL-C (<100 mg/dL), whereas 72% (36/50) of patients had elevated non-HDL-C (>130 mg/dL), suggesting a better predictive value of non-HDL-C than LDL-C for atherosclerosis.^47^

### Importance of non-HDL-C

***Consensus statement 2:*** *Non-HDL-C measures the cholesterol content of all atherogenic particles and has been shown with robust evidence to confer better estimation of ASCVD risk than LDL-C does, particularly in people with atherogenic dyslipidemia (high TGs and low HDL-C), commonly found in obesity, MetS, and T2DM.*

***Consensus statement 3:*** *Non-HDL-C is calculated from the lipid profile by subtracting the HDL-C from the total cholesterol (non-HDL-C = total cholesterol − HDL-C).*

***Consensus statement 4:*** *Evaluation of non-HDL-C should be performed particularly in patients with atherogenic dyslipidemia.*

***Consensus statement 5:*** *Evidence suggests that non-HDL-C is an important marker of CV risk and should be considered when assessing CV risk in patients with atherogenic dyslipidemia.*

***Consensus statement 6:*** *Non-HDL-C is recommended as a treatment target in patients with atherogenic dyslipidemia.*

***Consensus statement 7:*** *The non-HDL-C computation is less prone to errors than calculated LDL-C (Friedewald’s formula) in patients with elevated TGs.*

Rapid urbanization, high numbers of smokers, dietary changes, and increasingly sedentary lifestyles have fueled the development of dyslipidemia,^28^^,^^48^ obesity, MetS,^31^ T2DM, and hypertension in the APAC region, leading to increasing prevalence of CVD and associated mortality.^49^ Dyslipidemia is also a common comorbidity in patients with obesity,^50^^,^^51^ T2DM,^52^ and MetS;^53^ characterized by increased hepatic flux of free fatty acids (FAs) and elevated levels of TGs, small dense LDL, and ApoB, combined with low levels of HDL-C.^52^^,^^54^^,^^55^ This atherogenic dyslipidemia often presents with “normal” total cholesterol and LDL-C concentrations, and may therefore be overlooked and remain untreated, further aggravating atherosclerosis progression and CVD-related events.^15^ Non-HDL-C measurement offers considerable clinical advantages, particularly in patients with T2DM, MetS, and low LDL-C levels, and those who are overweight or obese, where LDL-C may underestimate the CV risk.^56^ In T2DM, a distinct dyslipoproteinemia is observed, characterized by elevated VLDL levels, reduced HDL-C, and altered particle distribution across all lipoprotein classes, further limiting the applicability of LDL-C in such cases.^56^ Elevated non-HDL-C can also serve as a potential predictor for MetS diagnosis.^57^ In patients with well maintained or low LDL-C, non-HDL-C remains a robust predictor of myocardial infarction (MI), ASCVD risk, and all-cause death.^18^ In patients with obesity, where TG-rich lipoproteins are often elevated, measuring non-HDL-C in addition to LDL-C is recommended for a comprehensive lipid profile assessment.^58^

### Non-HDL-C is an important CV risk marker and treatment target in dyslipidemia

Although LDL-C levels fail to account for all of the atherogenic lipid components, non-HDL-C encompasses the cholesterol content in all of the atherogenic particles, including VLDL, IDL, LDL, chylomicron remnants, and Lp(a).^15^ However, before recommending non-HDL-C as a primary risk marker or treatment target, it is crucial to ascertain its equivalence or superiority to LDL-C for CVD risk prediction.^59^

Evidence from meta-analyses^19^^,^^20^^,^^60^ and population studies has outlined non-HDL-C as a better CVD predictor than LDL-C.^61^, ^62^, ^63^, ^64^ Large meta-analyses have shown that increased levels of non-HDL-C are strongly associated with higher mortality risk in patients with or without coronary heart disease (CHD).^20^^,^^60^ One meta-analysis of 233,455 subjects estimated that using non-HDL-C as a CV risk marker and treatment target would prevent 300,000 more events over a decade than an LDL-C–centric approach.^19^ Recent cohort studies suggest that non-HDL-C is a useful marker for residual ASCVD risk in individuals at LDL-C goals and may provide additional prognostic information.^18^^,^^65^ In the Copenhagen General Population Study, elevated ApoB and non-HDL-C accounted for twice the proportion of ischemic strokes than with elevated LDL-C;^66^ furthermore, elevated ApoB and non-HDL-C (but not LDL-C) were associated with residual risk of all-cause mortality and MI in patients treated with statins.^21^ The EPIC (European Prospective Investigation Into Cancer and Nutrition)—Norfolk study, with a long-term follow-up (11 years) of 21,448 participants, reported that non-HDL-C was more strongly associated with the future risk of CHD than was LDL-C. Participants with elevated non-HDL-C levels (>130 mg/dL vs <130 mg/dL; HR: 1.84; 95% CI: 1.12-3.04), had a higher adjusted risk of future CHD, regardless of LDL-C levels.^67^

A strong association of non-HDL-C with CVD and related mortality has also been supported by studies from the APAC region.^65^^,^^68^ In Japan, the National Integrated Project for Prospective Observation of Non-Communicable Disease and Its Trends in the Aged, 1990 (NIPPON DATA 90) study, with 20 years of follow-up, demonstrated a strong association of non-HDL-C levels with the fatal coronary disease development.^69^ Furthermore, in patients with mild to moderate hypercholesterolemia with or without diabetes, increased non-HDL-C levels reflected a higher risk for CV events compared with LDL-C.^61^ The Cardiovascular Occupational Risk Factor Determination in Israel (CORDIS) study with 22-year follow-up of 4,832 patients, found that the non-HDL-C levels ≥190 mg/dL were more strongly associated with CVD mortality (HR: 1.80; 95% CI: 1.10-2.96) than LDL-C levels were (HR: 1.53; 95% CI: 0.98-2.39).^62^ Similarly, the on-treatment non-HDL-C, but not LDL-C levels, better predicted recurrent major adverse cardiovascular events (MACE) in patients with ASCVD with or without chronic kidney disease in the multicenter Taiwanese Secondary Prevention for Patients With Atherosclerotic Disease Registry (T-SPARCLE) study.^63^ Suzuki et al also reported that in statin-treated Japanese patients, elevated non-HDL-C (but not TG or LDL-C alone) was associated with a higher incidence of recurrent acute MI.^64^ A recent cohort study involving 3,866,366 Korean individuals reported that higher non-HDL-C levels were more strongly associated with a higher CV event risk (stroke and MI) than LDL-C levels were.^17^

### Non-HDL-C is less prone to estimation errors

Non-HDL-C provides a more comprehensive CV risk estimation than LDL-C, especially when TGs are elevated, a challenge increasingly encountered because of the rising prevalence of obesity, T2DM, and MetS.

LDL-C measurement by means of β-quantification (ultracentrifugation) provides accurate results, but its use in routine clinical practice is limited because it is time consuming and labor intensive.^70^ Another method of direct LDL-C measurement is the use of assays, but this may be challenging because of the lack of standardization across laboratories, high time consumption, and cost.^71^ Thus, LDL-C is routinely estimated with the use of the empirical Friedewald equation (calculated LDL-C).^70^ The limitations of using the Friedewald equation is its inaccuracy in conditions such as high TG levels (>4.52 mmol/L [>400 mg/dL]), low TG levels (<1.7 mmol/L [<150 mg/dL]), and very low levels of LDL-C (<1.8 mmol/L [<70 mg/dL]).^71^ Such low LDL-C levels are often attained when proprotein convertase subtilisin/kexin type 9 (PCSK9) inhibitors are added to statins.^71^

In contrast, non-HDL-C offers practical advantages such as evaluation under nonfasting conditions and no additional costs over conventional lipid tests.^23^^,^^72^ It is calculated as total cholesterol minus HDL-C. Thus, it accounts for the cholesterol in all atherogenic ApoB-containing lipoproteins.^23^^,^^72^ Using non-HDLC as a complementary target along with calculated LDL-C may also balance any under- or overestimation of LDL-C, owing to the uncertainty of the measurement or calculation at LDL-C values nearing 70 mg/dL (1.8 mmol/L).^15^ Unlike LDL-C, non-HDL-C is unaffected by TG level variability, making it a more reliable and robust measure for CVD risk assessment, especially in patients with hypertriglyceridemia.^15^^,^^23^^,^^72^

The advantages and disadvantages of non-HDL-C vs LDL-C are presented in the Central Illustration. The panel recommended that, for individuals with hypertriglyceridemia (>4.5 mmol/L), clinicians could treat the elevated TGs to a level <4.5 mmol/L and repeat the lipid profile to evaluate LDL-C, assess direct LDL-C if available and affordable, or use an alternative lipid target, such as non-HDL-C or ApoB. However, the panel recognized that direct LDL-C and ApoB measurements are often limited by affordability and availability concerns, and therefore, non-HDL-C may be the preferred parameter in the APAC region.

**Central Illustration:**
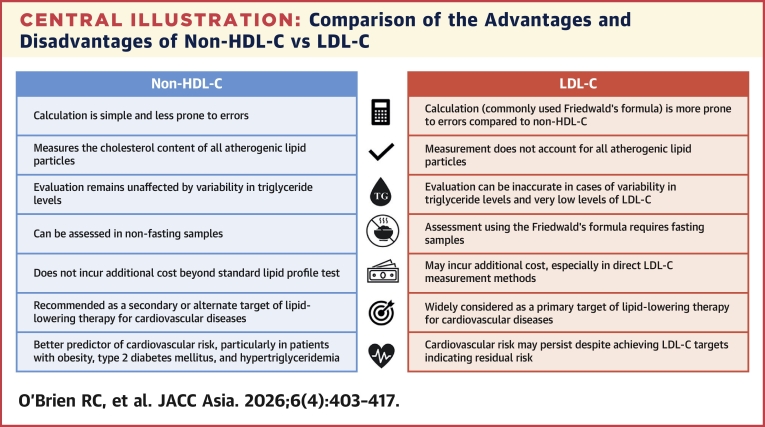
Comparison of the Advantages and Disadvantages of Non-HDL-C vs LDL-C Non-HDL-C (vs LDL-C) offers practical advantages, including ease of calculation, lower susceptibility to error, measurement under nonfasting conditions, and no additional cost beyond conventional lipid testing. In specific patient populations, non–HDL-C can be a better predictor of cardiovascular risk than LDL-C. LDL-C = low-density lipoprotein cholesterol; non-HDL-C = non-high-density lipoprotein cholesterol.

Owing to the susceptibility of LDL-C to inaccuracies in individuals with atherogenic dyslipidemia (obesity, MetS, and T2DM), hypertriglyceridemia, and very low LDL-C levels, routine non-HDL-C measurement is recommended for ASCVD risk estimation.^3^ Evidence showed non-HDL-C assays to be better than direct and calculated LDL-C methods in classifying individuals for ASCVD risk, regardless of TG levels, compared with their corresponding reference measurement procedures.^59^

Non-HDL-C is an important treatment target for CHD prevention; a linear relationship exists between its percentage reduction and CHD risk reduction with most lipid-lowering monotherapies, particularly statins and fibrates.^73^

Evidence from observational studies has also established a strong association of non-HDL-C with CV events. For example, in the Copenhagen General Population study, elevated levels of non-HDL-C accounted for a higher risk of all-cause mortality, MI, and ischemic stroke than did elevated LDL-C levels.^21^^,^^66^ Furthermore, higher non-HDL-C levels (≥5.7 mmol/L) were associated with 3 to 4 times higher CV event rates compared with lower non-HDL-C levels (<2.6 mmol/L) over 30 years (women: 33.7% vs 7.7%; men: 43.6% vs 12.8%).^74^

Although recent American and European guidelines, including the UK’s National Institute for Health and Care Excellence, identify non-HDL-C as a primary or co-primary target in dyslipidemia, it remains overlooked and is still a secondary target in most APAC guidelines.^5^^,^^33^, ^34^, ^35^, ^36^^,^^75^

### ApoB as an alternative lipid parameter

***Consensus statement 8:*** *ApoB is an alternative* test *to non-HDL-C, but it is more costly, is not widely available, and has limitations around international standardization.*

ApoB is a key structural component of chylomicrons, VLDL, IDL, LDL, and Lp(a).^23^ Accumulating evidence has shown that ApoB is superior to LDL-C and either better than or similar to non-HDL-C for CV risk assessment,^19^^,^^21^^,^^22^^,^^76^ and it is considered to be a secondary treatment target in CVD and hypertriglyceridemia.^3^^,^^15^ ApoB measurements are more accurate and reliable than those of LDL-C and non-HDL-C.^15^ Moreover, ApoB estimation does not require fasting and is easily automated,^15^ making it a promising alternative to non-HDL-C. However, lack of an appropriate primary reference standard and method renders the standardization of ApoB assays difficult.^15^^,^^23^ Furthermore, it is not widely available in general practice in many APAC countries and incurs additional costs over standard lipid tests. Therefore, considering affordability and availability, consensus recommends that non-HDL-C may be a more pertinent option than ApoB in the APAC region. Nonetheless, in cases of discordance between non-HDL-C and ApoB (such as when ApoB-containing particles are cholesterol enriched or cholesterol depleted), ApoB is a more reliable predictor than non-HDL-C of CV risk.^77^^,^^78^ Moreover, ApoB directly quantifies the number of atherogenic lipoproteins, whereas non-HDL-C reflects only the cholesterol content within these particles.^56^^,^^77^^,^^78^ Evidence indicates that CV risk is more strongly associated with ApoB particle concentration than to the mass of cholesterol within them.^77^^,^^78^ Findings from a discordance analysis suggested that ApoB is a more robust marker of CV risk compared to LDL-C, non-HDL-C, or triglycerides.^77^ In addition, unlike other routine cholesterol markers, ApoB measurement is automated and standardized.^79^ It enables identification of ApoB dyslipoproteinemias, including type III hyperlipoproteinemia, which cannot be accurately diagnosed with the use of cholesterol markers alone.^78^

### Non-HDL-C targets for the treatment of dyslipidemia

***Consensus statement 9:*** *For dyslipidemia treatment, the non-HDL-C target should be 30 mg/dL (0.8 mmol/L) higher than the desired LDL-C target.*

Major global guidelines recommend goals of lipid-lowering therapy, but there is heterogeneity in defining the targets. The American College of Cardiology/American Heart Association guidelines recommend a ≥50% LDL-C reduction for higher risk groups and specify either moderate- or high-intensity statin therapy, rather than setting targets.^80^ However, for secondary prevention, they do recommend LDL-C and non-HDL-C thresholds of 70 mg/dL (1.8 mmol/L) and 100 mg/dL (2.6 mmol/L), respectively, for the intensification of treatment with add-on therapies to statins.^80^ In contrast, the European guidelines recommend LDL-C goals based on the ASCVD risk profile and a secondary goal for non-HDL-C as 30 mg/dL (0.8 mmol/L) above the corresponding LDL-C goal, and that adjustment of lipid-lowering therapies is to be made in patients with very high risk CV based on the secondary goal (non-HDL-C) once the LDL-C goals are met.^3^ Specifically, for patients at very high, high, and moderate risk of CV, the recommended LDL-C goals, respectively, are <55 mg/dL (<1.4 mmol/L), <70 mg/dL (<1.8 mmol/L), and <100 mg/dL (<2.6 mmol/L); the respective non-HDL-C goals are <85 mg/dL (<2.2 mmol/L), <100 mg/dL (<2.6 mmol/L), and <130 mg/dL (<3.4 mmol/L).^3^ Most APAC guidelines base the non-HDL-C goals relative to the LDL-C levels (30 mg/dL above the LDL-C target).^6^^,^^7^^,^^9^^,^^81^ The consensus panel recommended that regardless of the approach to defining the targets, clinicians must initiate or intensify treatment for dyslipidemia if non-HDL-C levels are not at goal.

### Management of non-HDL-C

***Consensus statement 10:*** *In individuals with elevated non-HDL-C not at LDL-C goals, initial treatment should be to intensify LDL-C–targeted therapies (statin ± ezetimibe ± bile acid sequestrants ± bempedoic acid ± PCSK-9 inhibitors) according to availability.*

In individuals with elevated non-HDL-C and LDL-C, the initial treatment goal should be to achieve LDL-C targets based on risk classification. If the lipid targets are unmet with a maximally tolerated statin, add-ons of a cholesterol absorption inhibitor (ezetimibe),^82^ a bile acid sequestrant (colesevelam, colestimide, and cholestyramine),^83^, ^84^, ^85^ bempedoic acid (adenosine triphosphate-citrate lyase inhibitor),^86^^,^^87^ and a PCSK9 inhibitor should be considered.^88^^,^^89^

Given that the availability and affordability of medicines remain an important concern in many APAC countries,^90^^,^^91^ the consensus panel recommends that intensifying LDL-lowering therapies beyond statins should be guided by the availability and affordability of therapeutic options in each country.

***Consensus statement 11:*** *In individuals with elevated non-HDL-C with LDL-C at target, fibrates and some omega-3 FAs have been shown to reduce CV events.*

***Consensus statement 12:*** *The reduction in CVD risk appears to be proportional to the degree of non-HDL-C lowering, regardless of the type of therapy (diet or drug) (statins, fibrates, and bile acid sequestrants).*

***Consensus statement 13:*** *Omega-3 FAs may play a beneficial role in comprehensive lipid control and, possibly, additional reduction of CVD events in patients with atherogenic dyslipidemia; however, these benefits are largely confined to trials using high-dose pure EPA.*

***Consensus statement 14:*** *Fibrates may impart a beneficial role in comprehensive lipid control and, possibly, additional reduction of CVD events in patients with atherogenic dyslipidemia.*

***Consensus statement 15:*** *The combination of some fibrates, particularly gemfibrozil, with statins leads to an increased risk of myopathy owing to drug interactions; however, this has not been shown with fenofibrate, which makes it the preferred choice for combination with statins.*

Although statin monotherapy in atherogenic dyslipidemia reduces the risk of CV events, it does not eliminate the residual risk due to low HDL-C or high TG levels.^92^ Therefore, add-on therapies that reduce TGs, such as fibrates and omega-3 FAs, are used. In individuals who fail to achieve target lipid parameters with maximally tolerated doses of statins, add-on omega-3 FAs may improve the overall lipid profile,^93^ but their benefits for an additional reduction of CVD events in atherogenic dyslipidemia remain uncertain. Low-dose omega-3 FAs have shown no CV benefits,^94^^,^^95^ and limited evidence supports CV benefits at high doses.^96^^,^^97^ The REDUCE-IT (Reduction of Cardiovascular Events With Icosapent Ethyl—Intervention Trial) demonstrated that in patients with elevated TG levels, despite statin therapy, the omega-3 FA icosapent ethyl (4 g/d) lowered TG levels from baseline to 1 year (18.3% reduction vs 2.2% increase in placebo group) and significantly reduced CV events vs the mineral oil placebo.^96^ Similarly, the JELIS (Japan EPA Lipid Intervention Study) in patients with hypercholesterolemia demonstrated a significant reduction in TGs (9% vs 4%; *P* < 0.0001) and greater CV benefits with eicosapentaenoic acid (EPA; 1.8 g/d) added to statin therapy vs statin therapy alone.^97^ However, in the long-term outcomes from the STRENGTH (Statin Residual Risk With Epanova in High Cardiovascular Risk Patients With Hypertriglyceridemia) study showed no significant difference in the composite outcome of MACE with EPA and docosahexaenoic acid (DHA; 4 g/d) vs corn oil (inert comparator) in patients on statins with high CV risk, elevated TGs, and low HDL-C.^98^ Nevertheless, EPA/DHA significantly lowered TGs (−19.0% vs −0.9%), non-HDL-C (−6.1% vs −1.1%), and elevated HDL-C (5.0% vs 3.2%) vs the control group.^98^

The RESPECT-EPA Randomized Trial for Evaluation in Secondary Prevention Efficacy of Combination Therapy—Statin and Eicosapentaenoic Acid found that icosapentethyl treatment resulted in a numerically lower risk of CV events vs control in patients with chronic CAD on statin therapy, although it did not reach statistical significance.^99^

### Fibrate monotherapy

Fibrates, a class of peroxisome proliferator-activated receptor agonists, are known for their clinical effects on atherogenic dyslipidemia (ie, reducing TGs and elevating HDL-C).^100^ The Helsinki Heart Study showed CV risk reduction with gemfibrozil vs placebo in primary prevention individuals with elevated non-HDL-C.^101^ Gemfibrozil increased HDL-C and reduced LDL-C, non-HDL-C, and TGs vs placebo. However, the BIP (Bezafibrate Infarction Prevention) study^102^ and the LEADER (Lower Extremity Arterial Disease Event Reduction)^103^ trial demonstrated no overall CV risk reduction with bezafibrate monotherapy vs placebo. Nonetheless, a significant 39.5% reduction in the cumulative probability of fatal or nonfatal MI or death was observed in the subgroup of patients with elevated baseline TGs (≥200 mg/dL [≥2.3 mmol/L]), and a 41.8% reduction in the primary outcome was observed with the subgroup of patients with atherogenic dyslipidemia (elevated TGs and low HDL-C) with bezafibrate in the BIP study.^102^ Similarly, the DAIS (Diabetes Atherosclerosis Intervention Study) in patients with T2DM suggested reduced angiographic progression of CAD with fenofibrate,^104^ whereas the FIELD (Fenofibrate Intervention and Event Lowering in Diabetes) study showed no significant reduction in primary outcome (CHD death and nonfatal MI) of fenofibrate vs placebo.^105^ However, a prespecified analysis of patients with TG ≥200 mg/dL (2.3 mmol/L) and low HDL-C demonstrated a 27% reduction in CV events with fenofibrate.^106^

### Fibrate as an add-on therapy to statins

A registry-based analysis of 8,982 patients with ACS from the ACSIS (Acute Coronary Syndrome Israeli Survey) demonstrated that 30-day MACE risk after hospitalization due to ACS was remarkably lower with fibrate/statin combination treatment compared with statin monotherapy.^107^ In the ACCORD (Action to Control Cardiovascular Risk in Diabetes) trial, adding fenofibrate to statins in patients with diabetes failed to demonstrate overall CV benefit, but it reduced MACE in the subgroup with elevated TGs and low HDL-C levels at baseline.^108^ Notably, only 17% of the ACCORD study population had atherogenic dyslipidemia, a key indication for fibrate therapy, possibly explaining the lack of CV benefit in the overall population.^92^^,^^108^ Later, in the ACCORD follow-on study (ACCORDION), a post-trial 5-year follow-up of the ACCORD study, fenofibrate was associated with a 27% reduction in the primary outcome in patients with T2DM having elevated TGs and low HDL-C compared with statin monotherapy.^109^ The ECLIPSE-REAL (Effectiveness of Fenofibrate Therapy in Residual Cardiovascular Risk Reduction in the Real-World Setting) study also concluded that a statin plus fenofibrate therapy was associated with significantly reduced risk of composite CV events compared with statin monotherapy (adjusted HR [aHR]: 0.74; 95% CI: 0.58-0.93; *P* = 0.01) in Korean adult patients with MetS.^110^ Large meta-analyses suggested modest CV risk reduction in primary and secondary prevention populations with hypertriglyceridemia or atherogenic dyslipidemia who received fibrates.^111^, ^112^, ^113^ CHD risk reduction has been reported to be proportional to the magnitude of non-HDL-C lowering, by means of both diet and lipid-modifying drugs (statins, fibrates, and bile acid sequestrants),^73^^,^^114^ with a ∼1:1 relationship observed between the percentage of non-HDL-C lowering and CHD reduction.^73^

Whether all fibrates have similar effects on CV risk in dyslipidemia remains uncertain. The PROMINENT (Pemafibrate to Reduce Cardiovascular Outcomes by Reducing Triglycerides in Patients With Diabetes) trial showed that in 10,497 patients with T2DM having mild to moderately elevated TG, low HDL-C, and low LDL-C levels, pemafibrate therapy improved lipid profile by lowering TG, VLDL cholesterol, remnant cholesterol, and apolipoprotein C-III levels; however, LDL-C and ApoB levels increased, non-HDL-C was unchanged, and the risk of CV events was not reduced.^115^

Although fibrates aid in improving the atherogenic lipid profile and CV risk reduction when added to the statins, the combination of some fibrates with statins may cause myopathy. The risk of myopathy, mostly reported with the use of gemfibrozil, is related to pharmacokinetic interaction: Gemfibrozil inhibits the metabolic glucuronidation of the statin, leading to elevations in the plasma levels of statins.^116^ Fenofibrate has a different pattern of uridine diphosphate-glucuronosyltransferase enzyme selectivity for glucuronidation compared with gemfibrozil and the statins; resulting in less interference with statin metabolism and a lower myopathy incidence.^117^^,^^118^ Global^119^^,^^120^ and regional^121^, ^122^, ^123^, ^124^ studies support efficacy (improvement of lipid profile) and safety (risk of myopathy) of fenofibrate when combined with various statins. The U.S. Food and Drug Administration’s Adverse Event Reporting System found that the reports of rhabdomyolysis per million prescriptions dispensed were much lower for fenofibrate than for gemfibrozil in combination with statins (4.5 vs 87).^125^ A meta-analysis reported that the statin and fenofibrate combination had a safety profile similar to that of statin monotherapy.^126^ Moreover, fenofibrate has shown better efficacy than gemfibrozil in improving the lipid profile, both as monotherapy and with statins.^127^^,^^128^ There are very limited data from small studies to evaluate the risk of myopathy with bezafibrate.^129^, ^130^, ^131^ Given the efficacy and safety evidence, the consensus panel recommended fenofibrate as the preferred fibrate to be used in combination with statins.

***Consensus statement 16:*** *Fenofibrate therapy has been shown to lower the risk of microvascular complications in patients with T2DM, particularly with robust evidence for retinopathy and microvascular-related amputation; however, its renal benefits need further confirmation.*

***Consensus statement 17:*** *In patients with elevated TGs and low HDL-C, fenofibrate add-on to the ongoing statin treatment may reduce the risk of macrovascular (cardiovascular and cerebrovascular) complications, particularly in those with T2DM or MetS.*

### Fenofibrate and the risk of microvascular complications in patients with type 2 diabetes

Several randomized and observational studies have shown that fenofibrate, as monotherapy or with statins, reduces the risk of diabetic retinopathy and may offer other microvascular benefits in patients with T2DM.^132^, ^133^, ^134^, ^135^, ^136^ In the FIELD study, fenofibrate treatment (vs placebo) significantly reduced the risk of microvascular complications, particularly retinopathy (requirement for first laser treatment, 3.4% vs 4.9%; *P* = 0·0002).^136^^,^^137^ In the ACCORD trial substudy (the ACCORD Eye Study), fenofibrate (as an add-on to statin) significantly reduced diabetic retinopathy progression compared with placebo (6.5% vs 10.2%; *P* = 0.006) in patients with T2DM.^132^ Recently, the LENS (Lowering Events in Nonproliferative Retinopathy in Scotland) trial assessed fenofibrate monotherapy (vs placebo) on the progression of diabetic retinopathy in patients with nonreferable diabetic retinopathy or maculopathy over a median follow-up of 4.0 years (Q1-Q3: 3.6-4.3 years). Fenofibrate was associated with approximately 27% lower risk of progression of referable diabetic retinopathy or maculopathy or their treatment (including intravitreal injection of medication, retinal laser therapy, or vitrectomy) compared with placebo: HR: 0.73; 95% CI: 0.58-0.91; *P* = 0.006. Of note, the benefits of fenofibrate were observed across patients with type 1 and type 2 diabetes and in those with normal and mild to moderate renal impairment.^138^

These observations from randomized trials corroborate reports of cohort studies in the Asian population. A propensity-matched cohort study in Korean patients with T2DM and MetS on statin therapy concluded that fenofibrate as an add-on to statins (vs statin monotherapy) was associated with a significantly lower risk of diabetic retinopathy progression including vitreous hemorrhage, laser photocoagulation, and intravitreal injection therapy.^133^ Similarly, a retrospective cohort study with a total follow-up period of 6.8 ± 1.5 years for fenofibrate users (n = 2,500) and 5.4 ± 2.6 years for nonusers (n = 29,753) demonstrated a lower risk of incident retinopathy (HR: 0.57 [95% CI: 0.57-0.62; *P* < 0.001]; aHR: 0.75 [95% CI: 0.68-0.82; *P* < 0.001]) and need for laser treatment (HR: 0.59 [95% CI: 0.49-0.71; *P* < 0.001]; aHR: 0.67; 95% CI: 0.56-0.81; *P* < 0.001]) with fenofibrate.^134^

Furthermore, in the FIELD study, patients who received fenofibrate vs placebo were found to have a 36% lower risk of first nontraumatic amputation (HR: 0.64; 95% CI: 0.44-0.94; *P* = 0.02) and 47% lower risk of minor amputations without known large-vessel disease (HR: 0.53; 95% CI: 0.30-0.94; *P* = 0.027).^139^

The renal benefits of fenofibrate have been observed in studies such as FIELD and ACCORD. In the FIELD substudy involving patients with T2DM, fenofibrate vs placebo significantly reduced albuminuria and lower estimated glomerular filtration rate decline over 5 years, despite an initial reversible rise in plasma creatinine.^140^ In the ACCORD study, the incidence of both micro- and macroalbuminuria was significantly lower in patients receiving fenofibrate (an add-on to statin) vs placebo.^108^ However, more concrete evidence is still needed to confirm the benefits of fenofibrate therapy in reducing nephropathic complications in patients with T2DM.

***Consensus statement 18:*** *In patients with atherogenic dyslipidemia, a combination of statin and fenofibrate offers a reduction in non-HDL-C greater than with statin monotherapy.*

Fenofibrate as an add-on to statin therapy has shown improvements in the lipoprotein profile, particularly in atherogenic dyslipidemia. A study involving patients with MetS having combined hyperlipidemia demonstrated that adding fenofibrate to simvastatin significantly decreased total TGs (−38%), VLDL + IDL-C (−36%), and VLDL + IDL-ApoB (−34%) and increased HDL-C levels (16%) compared with statin-only therapy.^141^ In the SAFARI (Simvastatin Plus Fenofibrate for Combined Hyperlipidemia) trial, fenofibrate plus simvastatin resulted in better improvements in the lipoprotein profile and a greater reduction in non-HDL-C than simvastatin monotherapy (−35.3% vs −26.1%).^119^ A similar trend was observed in a randomized study evaluating fenofibrate/pravastatin combination therapy in high-CV-risk subjects with mixed hyperlipidemia that was not controlled on pravastatin monotherapy. At week 12, the combination therapy showed a greater reduction in non-HDL-C compared with statin monotherapy (14.1% vs 6.1%). After 64 weeks of treatment, combination therapy significantly reduced non-HDL-C (14.1%), LDL-C (10.2%), and ApoB (9.3%), along with an elevation in HDL-C (4.7%) and apoA1 (10.3%; all *P* < 0.001) compared with baseline.^142^ Similarly, pitavastatin/fenofibrate combination showed greater non-HDL-C reduction and lipid profile improvement than pitavastatin alone in Korean patients with mixed dyslipidemia at high CVD risk.^122^

Treatment targeting non-HDL-C ensures comprehensive CV risk reduction, particularly in patients with T2DM, MetS, hypertriglyceridemia, and low HDL-C, where LDL-C alone may underestimate the risk. Future treatment paradigms should consider incorporating non-HDL-C as a co-primary lipid target to address the residual cardiovascular risk that persists even after achieving optimal LDL-C levels, because non-HDL-C more accurately reflects the overall atherogenic burden.

## Conclusions

This consensus from a diverse group of clinical experts from the APAC region, based on available evidence and clinical practice, fills the gap for a practical clinical guidance on adopting non-HDL-C as a lipid parameter in atherogenic dyslipidemia management. Add-on therapies such as omega-3 FAs and fibrates (particularly fenofibrate) to statins are recommended for the reduction of non-HDL-C and CV risk in patients with atherogenic dyslipidemia commonly observed in conditions such as obesity, T2DM, and MetS.

## Funding Support and Author Disclosures

Indegene received funding from Abbott for development of this article. Dr O’Brien is a member of the board, advisory board, or drug safety monitoring board for Abbott, Amgen, AstraZeneca, Eli Lilly, MSD, Novartis, Novo Nordisk, and Sanofi; has received honoraria from Abbott, Amgen, AstraZeneca, Boehringer Ingelheim, Eli Lilly, Novartis, and Novo Nordisk; and serves in a leadership or fiduciary role as executive member, immediate past president of Asia Pacific Society of Atherosclerosis and Vascular Diseases, Lipid Clinic Coordinator, Asia Pacific Region of European Atherosclerosis Society, and Clinical Council Member of Australian Atherosclerosis Society. Dr Tomlinson has received honoraria from Abbott, Amgen, and Novartis. Dr Nawawi is a member of the board, advisory board, or drug safety monitoring board for Abbott, Amgen, and Sanofi; has received honoraria from Amgen and Novartis; and has received research funding from Amgen. Dr Ong is a member of the board, advisory board, or drug safety monitoring board for Moderna and has received honoraria from DKSH and Viatris. Dr Silki was an employee of Indegene, India, at the time the study was conducted. Dr Vinh is a member of the board, advisory board, or drug safety monitoring board for AstraZeneca, Boehringer Ingelheim, Roche Diagnostics, and Servier; has received honoraria from Abbott, AstraZeneca, Boehringer Ingelheim, Novartis, Novo Nordisk, Menarini, PharmaNord, Pfizer, Roche Diagnostics, Sanofi, Servier, United Pharma, and Daiichi-Sankyo; and has received other support (eg, trips, travel, equipment, materials, drugs, medical writing, gifts, and other services) from AstraZeneca, Boehringer Ingelheim, GE Healthcare, MSD, Philips Servier, and United Pharma/Vietnam. The other authors have no relationships relevant to the contents of this paper to disclose.
