# Supplementary material for: Importance and Management of Non–High-Density Lipoprotein Cholesterol in Dyslipidemia Treatment
Source: JACC Asia. 2026 Apr 7;6(4):403–17. doi: 10.1016/j.jacasi.2025.12.024 (PMC13080752; doi:10.1016/j.jacasi.2025.12.024)
Supplement: Supplemental Data [file mmc1.docx]

**Supplemental Methods**

The study was conducted in four key steps: (i) formation of a core committee, literature search, and development of the statements; (ii) selection of a panel comprising specialists representative of the APAC region; (iii) voting on the statements by the panel through an online/web-based survey; and (iv) analysis and discussion on the voting results by the expert panel **(Figure 1)**.

Panel members were selected based on their clinical and scientific expertise and practice concerning dyslipidemia management and were representative of the APAC region. The panel consisted of 11 clinicians with recognized academic and clinical expertise, with 25 to >50 years of practice and expertise in internal medicine, cardiology, endocrinology, and metabolic medicine. The core group consisted of three experts from Australia, Hong Kong, and the Philippines **(Supplemental Table 3)**.

A web-based survey was designed to capture the agreement of experts on the proposed statements with objective response options (“agree,” “disagree,” and “agree with modifications”). A free text option allowed respondents to provide comments to substantiate the responses. The survey was administered along with the relevant literature via email in May 2024 to the experts previously invited to participate in the study. Consensus was achieved when ≥80% of the experts agreed with the statements. In case of a disagreement, the consensus was achieved by a second round of voting on the modified statements or, if needed, through discussions during the consensus meeting.

A virtual consensus meeting was conducted on May 10, 2024, via Microsoft Teams, and the experts discussed the voting results and comments received during the voting to achieve a general agreement and formulate the final consensus statements. This was followed by the development of the consensus document that was approved by all the experts **(Figure 1)**.

**Supplemental Table 1** Search criteria for the comprehensive literature search

| **Databases** | PubMed |
| --- | --- |
| **Year filter** | None |
| **Search terms/keywords** |  |
| Dyslipidemia/atherogenic dyslipidemia | (((Dyslipidemia[MeSH Terms]) OR (dyslipidemia[Title/Abstract])) OR (Atherogenic dyslipidemia[Title/Abstract])) OR (Atherogenic dyslipidemia[Text Word]) |
| Residual risk, marker, and treatment target | (((((Residual risk[Text Word]) OR ("Biomarkers"[Mesh])) OR (marker[Text Word])) OR (treatment target[Text Word])) OR (treatment goal[Text Word])) OR (risk factor[Text Word]) |
| CVD | ((((((Cardiovascular disease[MeSH Terms]) OR ("Vascular Diseases"[Mesh])) OR (atherosclerotic cardiovascular disease[Title/Abstract])) OR (ASCVD[Title/Abstract])) OR (CV risk[Text Word])) OR ("Myocardial Ischemia"[Mesh])) OR ("Arteriosclerosis"[Mesh]) |
| Lipid parameters | (((((((low density lipoprotein cholesterol[MeSH Terms]) OR (low density lipoprotein cholesterol[Text Word])) OR (LDL-C[Text Word])) OR (non-high density lipoprotein cholesterol[Text Word])) OR (non-HDL-C[Text Word])) OR (apolipoprotein B[MeSH Terms]))) OR (ApoB[Text Word]) |
| Statins | (((((("Hydroxymethylglutaryl-CoA Reductase Inhibitors"[Mesh]) OR (Statin[Text Word])) OR (atorvastatin[Text Word])) OR (simvastatin[Text Word])) OR (lovastatin[Text Word])) OR (fluvastatin[Text Word])) OR (rosuvastatin[Text Word]) |
| Fibrates | ((((((("Fibric Acids"[Mesh]) OR (Fibrate[Text Word])) OR (fenofibrate[Text Word])) OR (bezafibrate[Text Word])) OR (Gemfibrozil[Text Word])) OR (clofibrate[Text Word])) OR (clofenapate[Text Word])) OR (pemafibrate[Text Word]) |
| Omega-3 FAs | (((Fish Oils[MeSH Terms]) OR (Fatty Acids, Omega-3[MeSH Terms])) OR (Eicosapentaenoic Acid[MeSH Terms])) OR (Eicosapentaenoic Acid[Text Word]) |
| Other lipid therapies | (((ezetimibe[Text Word]) OR (bempedoic acid[Text Word])) OR (bile acid sequestrants[Text Word])) OR (PCSK9 inhibitors[Text Word]) |
| Other filters | Language: English  Article type: Clinical trial, meta-analysis, randomized controlled trial, and observational study  Species: Human |
| Apo, apolipoprotein; ASCVD, atherosclerotic cardiovascular disease; CV, cardiovascular; CVD, cardiovascular disease; LDL-C, low-density lipoprotein cholesterol; non-HDL-C, non-high-density lipoprotein cholesterol; PCSK-9, proprotein convertase subtilisin/kexin type 9. | |

**Supplemental Table 2** Consensus statements on non-HDL-C

| **No.** | **Statement** |
| --- | --- |
| Importance of non-HDL-C | |
|  | Despite achieving the LDL-C target, the residual risk of ASCVD persists in patients with dyslipidemia, even after adjustment for other risk factors. This may be attributed to other atherogenic lipoproteins including Lp(a) and triglyceride-rich particles. |
|  | Non-HDL-C measures the cholesterol content of all atherogenic particles and has been shown with robust evidence to confer better estimation of ASCVD risk than LDL-C, particularly in people with atherogenic dyslipidemia (high triglycerides and low HDL-C), commonly found in obesity, metabolic syndrome, and diabetes. |
|  | Non-HDL-C is calculated from the lipid profile by subtracting the HDL-C from the total cholesterol (non-HDL-C = total cholesterol − HDL-C). |
|  | Evaluation of non-HDL-C should be performed particularly in patients with atherogenic dyslipidemia. |
|  | Evidence suggests that non-HDL-C is an important marker of CV risk and should be considered when assessing CV risk in patients with atherogenic dyslipidemia. |
|  | Non-HDL-C is recommended as a treatment target in patients with atherogenic dyslipidemia. |
|  | The non-HDL-C computation is less prone to errors than calculated LDL-C (using Friedewald’s formula) in patients with elevated triglycerides. |
|  | ApoB is an alternative test to non-HDL-C but it is more costly, not widely available, and has limitations towards international standardization. |
| Non-HDL-C as a treatment target in dyslipidemia | |
|  | For dyslipidemia treatment, the non-HDL-C target should be 30 mg/dL (0.8 mmol/L) higher than the desired LDL-C target. |
| Management of non-HDL-C | |
|  | In individuals with elevated non-HDL-C not at LDL-C goals, initial treatment should be to intensify LDL-C targeted therapies (statin ± ezetimibe ± bile acid sequestrants ± bempedoic acid ± PCSK-9 inhibitors) as per availability. |
|  | In individuals with elevated non-HDL-C with LDL-C at target, fibrates and some omega-3 FAs have been shown to reduce CV events. |
| Difference between fibrates: Are all fibrates the same? | |
|  | The reduction in CVD risk appears to be proportional to the degree of non-HDL-C lowering, regardless of the type of therapy (diet or drug) (statins, fibrates, and bile acid sequestrants). |
|  | Omega-3 FAs may play a beneficial role in comprehensive lipid control and, possibly, additional reduction of CVD events in patients with atherogenic dyslipidemia; however, these benefits are largely confined to the trials using high-dose pure EPA. |
|  | Fibrates may impart a beneficial role in comprehensive lipid control and, possibly, additional reduction of CVD events in patients with atherogenic dyslipidemia. |
|  | The combination of some fibrates, particularly gemfibrozil, with statins leads to an increased risk of myopathy due to drug interactions; however, this has not been shown with fenofibrate, which makes it the preferred choice for combination with statins. |
|  | Fenofibrate therapy has been shown to lower the risk of microvascular complications in patients with type 2 diabetes, particularly with robust evidence for retinopathy and microvascular-related amputation; however, its renal benefits need further confirmation. |
|  | In patients with elevated TGs and low HDL-C, fenofibrate add-on to the ongoing statin treatment may reduce the risk of macrovascular (cardiovascular and cerebrovascular) complications, particularly in those with type 2 diabetes or metabolic syndrome. |
|  | In patients with atherogenic dyslipidemia, a combination of statin and fenofibrate offers a reduction in non-HDL-C greater than statin monotherapy. |

Apo, apolipoprotein; ASCVD, atherosclerotic cardiovascular disease; CV, cardiovascular; CVD, cardiovascular disease; EPA, eicosapentaenoic acid; FA, fatty acid; HDL-C, high-density lipoprotein cholesterol; LDL-C, low-density lipoprotein cholesterol; non-HDL-C, non-high-density lipoprotein cholesterol; PCSK-9, proprotein convertase subtilisin/kexin type 9; TG, triglyceride; T2DM, type 2 diabetes mellitus.

**Supplemental Table 3** Details of the expert panel

| **Sr. No.** | **Name** | **Country** | **Specialization** | **Clinical experience (Years)** | **Role/ Contribution** |
| --- | --- | --- | --- | --- | --- |
|  | Richard O’Brien | Australia | Internal Medicine and Endocrinology | >35 | Consensus Chairperson, Core/Writing group |
|  | Lourdes Ella Gonzalez-Santos | Philippines | Medicine and Cardiology | 30 | Core/Writing group |
|  | Brian Tomlinson | China | Internal Medicine | 50 | Core/Writing group |
|  | Zanariah Hussein | Malaysia | Endocrinology | 33 | Consensus Panelist/Manuscript Review |
|  | Soo Lim | South Korea | Medicine | 30 | Consensus Panelist/Manuscript Review |
|  | Hapizah Nawawi | Malaysia | Chemical Pathology and Metabolic Medicine | >35 | Consensus Panelist/Manuscript Review |
|  | Hean Yee Ong | Singapore | Cardiology | 30 | Consensus Panelist/Manuscript Review |
|  | Sidartawan Soegondo | Indonesia | Endocrinology | >40 | Consensus Panelist/Manuscript Review |
|  | Ta-Chen Su | Taiwan | Internal Medicine and Cardiovascular | 28 | Consensus Panelist/Manuscript Review |
|  | Apichard Sukonthasarn | Thailand | Medicine | >45 | Consensus Panelist/Manuscript Review |
|  | Pham Nguyen Vinh | Vietnam | Cardiology | >50 | Consensus Panelist/Manuscript Review |
